# Prevalence of sleep disturbance and its associated factors among diabetes type-2 patients in Saudi Arabia

**DOI:** 10.3389/fpubh.2024.1283629

**Published:** 2024-11-01

**Authors:** Faisal Saeed Al-Qahtani

**Affiliations:** Department of Family and Community Medicine, College of Medicine, King Khalid University, Abha, Saudi Arabia

**Keywords:** diabetes, sleep disturbance, apnea, sleep medications, Saudi Arabia

## Abstract

**Introduction:**

This study investigated the prevalence of sleep disturbances among people living with type 2 diabetes in Saudi Arabia.

**Methods:**

A four-stage sampling method was used to recruit 479 participants from various parts of the country. A self-administered questionnaire was used to collect demographic, sleep disruption, and related clinical characteristics. Data analysis included reporting sleep disturbance prevalence as frequency and percentage, chi-square tests to examine categorical variable associations, and multiple logistic regression analysis to identify independent factors associated with sleep disturbances.

**Results:**

The data showed that most participants were male (59.3%), married (78.5%), and Diploma/ Bachelor’s degree holders (41.5%). The study found that most respondents had subjective sleep quality issues, with 55.3% of Sleep Latency participants having trouble falling asleep, 62.4% having trouble sleeping, 65.1% experiencing serious sleep efficiency impairment, 85.2% reporting sleep difficulties, 88.5% using sleep medication without difficulty, 5.4% having moderate difficulty, and 6.1% having considerable difficulties. The Global Pittsburgh Sleep Quality Index (PSQI) showed that all participants had sleep problems. Marital status, nationality, HBA1C, education, region, comorbidity, and monthly income were significantly associated with sleep disturbance characteristics.

**Conclusion:**

The study concluded that diabetes itself can increase the risk of sleep problems, as it is associated with various sleep-related issues such as insomnia, sleep apnea, and restless legs syndrome. Encouraging social support within the realm of healthcare services holds significance in reducing the occurrence of inadequate sleep. For individuals diagnosed with type 2 diabetes mellitus, managing their blood sugar levels effectively is crucial for enhancing the quality of their sleep. Furthermore, engaging in regular physical activity is essential for preventing subpar sleep quality.

## 1 Introduction

Diabetes mellitus is a metabolic condition caused by dysfunctions in the metabolism of carbohydrates, fats, and proteins, and is increasing due to aging populations, urbanization, sedentary lifestyles, and obesity (1). Diabetes affects an estimated 9.3% of the world’s population, with projections showing a rise to 10.2% by 2030 and 10.9% by 2045 (2). Sleep is essential for maintaining optimal bodily functions and overall health, as it facilitates the glands to produce their respective hormones that regulate metabolic and endocrine functions daily (3). Poor sleep quality is a health issue for everyone experiencing it, characterized by difficulty initiating and maintaining sleep, excessive drowsiness, disruptions in sleep–wake schedules, and dysfunctions related to sleep stages. It is associated with higher rates of inadequate sleep, daytime drowsiness, and increased use of sleep medications. Type 2 diabetes is a chronic metabolic disorder that affects millions of people worldwide and is increasing at an alarming rate (4)

Sleep disturbance is a common problem among individuals with type 2 diabetes, which can have a significant impact on their health and quality of life. Poor sleep quality and duration has been linked to various health problems, but the underlying mechanisms are not yet fully understood (5). Poor sleep quality in individuals with diabetes mellitus can have negative consequences, such as non-adherence to recommended medication, insulin resistance, cardiovascular disease, cognitive impairment, decreased working capacity, and a higher risk of stroke and depression (6, 7). Poor sleep quality in DM patients is linked to various factors, such as age, gender, body mass index, noise, and smoking/drinking habits (8). Therefore, it is essential to address these factors in order to improve sleep quality and overall health outcomes in individuals with DM (9, 10).

Various studies have been conducted in Saudi Arabia related to sleep disturbance and their related findings and the prevalence of sleep duration among Saudi adults (11–17). Few studies have been conducted in the Saudi population assessing sleep problems in diabetic patients (1, 14, 18). A comprehensive study is needed to investigate the prevalence of sleep disturbance and its associated factors among people with type 2 diabetes in Saudi Arabia. This study could help identify potential risk factors and inform the development of targeted interventions to improve the quality of life of individuals with type 2 diabetes. This study aims to investigate the prevalence of sleep disturbance and its contributing factors among people with type 2 diabetes in Saudi Arabia. The findings will provide insights for healthcare providers to develop interventions to improve sleep quality and overall health outcomes, potentially influencing diabetes management policies and guidelines in the country.

## 2 Methodology

### 2.1 Study design and population

The study design was a cross-sectional questionnaire-based conducted to investigate the prevalence of sleep disturbances and their associated factors among patients living with diabetes across all regions of Saudi Arabia. The study population included patients living with type 2 diabetes.

### 2.2 Sample size, sampling method, and data collection

The sampling technique used was a three-stage (multi-stage) sampling technique, with participants divided into four clusters based on the different regions of the kingdom and stratified into several strata based on their characteristics. Saudi Arabia features among the 21 countries and territories of the IDF MENA region. There are currently 537 million individuals worldwide who are affected by diabetes, with 73 million of them residing in the MENA Region. It is projected that by 2045, this number will increase to 135.7 million. As per 2021 data, the prevalence rate of diabetes mellitus in adults was 17.7%, with an estimated 4,274,100 total cases of diabetes in adults.

The final minimum estimated sample size of 385 participants was determined using the Raosoft sample size calculator.^1^ We assumed a precision level of 5% and a margin of error of 5%. Additionally, a confidence level of 95% was chosen to ensure the desired level of statistical significance. Data collection was conducted using an electronic self-entered questionnaire to collect demographic information (such as gender, age, and residence), clinical characteristics, information related to the sleep patterns including the Pittsburgh Sleep Quality Index (PSQI) Scoring (Supplementary File 2).

### 2.3 Construction, validation and reliability of questionnaire

To design the initial version of the questionnaire, an in-depth review of the pertinent literature (1–14) was conducted. Following this, specialists in the areas of community and family medicine, internal medicine, and epidemiology were consulted to ensure the questionnaire’s content validity. The valuable input and feedback from these professionals were carefully considered during the thorough revision process. In order to ensure the reliability of the study tool, a pilot study was carried out with a sample size of 30 individuals. The findings from the pilot study were incorporated into the final data analysis. In addition, the questionnaire’s reliability was evaluated by calculating the Cronbach’s alpha coefficient. The Cronbach’s alpha value of 0.72 suggests that the questionnaire has a satisfactory level of reliability.

### 2.4 Inclusion and exclusion criteria

The inclusion criteria included patients aged 18 years or older living with type 2 diabetes in Saudi Arabia, who can read and understand Arabic, and who provided informed consent. The exclusion criteria included patients who had any severe medical conditions or complications that interfere with their ability to complete the survey. The study excluded individuals with severe mental health disorders, those without diabetes, patients with acute medical conditions that could disrupt sleep patterns (such as recent surgery or acute infections), and pregnant women.

Definition of operational variables: The following definitions were used for the operational variables of the study.

#### 2.4.1 Sleep latency

Sleep latency refers to the amount of time it takes for a person to fall asleep after getting into bed. It is commonly used as a measure of sleep onset or sleep initiation.

#### 2.4.2 Sleep efficiency

Sleep efficiency is a measure that quantifies the quality and effectiveness of sleep. It represents the percentage of time spent asleep out of the total time spent in bed.

#### 2.4.3 Habitual sleep efficiency

Habitual sleep efficiency refers to the average sleep efficiency over an extended period of time, typically assessed across multiple nights or weeks. It provides an overall measure of how efficiently an individual sleeps on a regular basis.

#### 2.4.4 Subjective sleep quality

Subjective sleep quality refers to an individual’s personal assessment or perception of the quality of their sleep. It involves self-reporting and subjective judgments about various aspects of sleep, such as how easily they fall asleep, how restful their sleep feels, and how refreshed they feel upon awakening.

#### 2.4.5 Daytime dysfunction

Daytime dysfunction refers to difficulties or impairments experienced by an individual during their waking hours as a result of poor sleep or sleep-related issues. It includes a range of symptoms and problems that can interfere with a person’s daytime functioning, well-being, and overall quality of life.

#### 2.4.6 Sleep disturbances

Sleep disturbances refer to any disruptions or abnormalities in the normal sleep pattern. There are various types of sleep disturbances, including difficulties falling asleep, staying asleep, or experiencing poor-quality sleep.

### 2.5 Statistical analysis

The survey conducted among all diabetic patients living in Saudi Arabia was primarily categorical and the prevalence of sleep disturbances was reported as frequency and percentage. To investigate the factors that were associated with sleep disturbances in diabetic patients, a chi-square test and multiple logistic regression analysis were conducted.

### 2.6 Ethical

The Research Ethics Committee at King Khalid University reviewed and approved the research proposal, taking into account the potential risks and benefits to participants as well as the study’s overall scientific merit and integrity. All participants were apprised of the purpose and procedures of the study, and written informed consent was obtained. All participant information was kept confidential throughout the duration of the study.

## 3 Results

### 3.1 Demographic characteristics

Table 1 shows demographic characteristics. Age is divided into four categories ranging from 18 to 97 years old, with the largest percentage (51.1%) falling in the 38–57 years range. The majority of the participants were male (59.3%) and married (78.5%). Regarding education, the majority had Diploma/Bachelor’s degree education (41.5%). The study’s participants were drawn from diverse geographical regions, with the Southern region having the largest representation, accounting for 40.9% of the total sample. This region was selected due to the presence of a diabetic clinic that the participants were attending for their diabetes management. The majority of participants were Saudi (97.9%), with a large portion having a monthly income of 5,000 to 15,000 Saudi Riyals (35.1%).

**Table 1:** Demographic characteristics (*n* = 479).

Variable		Frequency	Percentage
Age	18–37 Years	132	27.6
	38–57 years	245	51.1
	58–77 years	96	20
	78–97 years	6	1.3
Gender	Male	284	59.3
	Female	195	40.7
Marital status	Single	65	13.6
	Divorced/widowed	38	7.9
	Married	376	78.5
Education	Basic education (intermediate primary or secondary education)	191	39.9
	Illiterate (cannot read or write)	58	12.1
	Diploma or bachelor’s degree postgraduate	19,931	41.56.5
	Postgraduate University education (diploma or bachelor’s degree)	31,199	6.541.5
Region	Central and Eastern Region (Riyadh and Eastern Regions)	110	23
	Southern region (Aseer, Al-Baha, Najran and Jazan regions)	196	40.9
	The northern region (Qassim, Al-Jawf, Hail, Tabuk and the northern border regions)	60	12.5
	Western region (Makkah and Madinah regions)	113	23.6
Nationality	Non-Saudi	10	2.1
	Saudi	469	97.9
Monthly income (Saudi Riyals)	I have no income and depend on others to spend	113	23.6
	Less than 5,000	113	23.6
	5,000–15,000	168	35.1
	More than 15,000	85	17.7

### 3.2 Clinical characteristics

Table 2 shows the clinical characteristics of the study participants. Diabetes duration varied, with the majority (53.2%) having more than 10 years of diabetes duration. Most of respondents (42.8%) had their recent glycosylated hemoglobin test (HBA1C) values from 7 to less than 9%. The highest frequency is for Diabetes (41.1%), followed by Diabetes + hypertension (23.2%), and Diabetes + Dyslipidemia (22.6%).

**Table 2:** Clinical characteristics.

Variable		Frequency	Percentage
Diabetes duration	less than 5 years	147	30.7
	From 5 to 10 years	77	16.1
	More than 10 Years	255	53.2
Glycosylated hemoglobin test (HBA1C)	I do not know	21	4.4
	Less than 7%	153	31.9
	From 7 to less than 9%	205	42.8
	From 9 to less than 11%	66	13.8
	More than 11%	34	7.1
Comorbidity	Diabetes only	197	41.1
	Diabetes + HTN (High Blood Pressure)	111	23.2
	Diabetes + Dyslipidemia	108	22.6
	Diabetes + Hypertension+ Dyslipidemia	17	3.5
	Other comorbidities	184	38.5

### 3.3 Usual sleeping habits

The majority (44.5%) of participants reported going to bed between 12:00 AM - 5:59 AM, followed by 30.5% who reported sleeping between 6 PM and 11:59 PM. The majority (42.8%) of participants reported taking 1–15 min to fall asleep, followed by 35.1% who took 16–30 min to fall asleep. The majority (55.7%) of participants reported getting up between 6 AM and 11:59 AM, followed by 12:00–5:59 AM (35.3%). The majority (32.6%) of participants reported sleeping for 5–6:59 h per night (Table 3).

**Table 3:** Usual sleep habits.

Questions		Frequency	Percent (%)
During the past month when you usually go to bed at night	12:00–5:59 AM	213	44.5
	6–11:59 AM	111	23.2
	12–5:59 PM	9	1.9
	6–11:59 PM	146	30.5
How long does it usually take you to fall asleep each night? (in minutes)	1–15 Minutes	205	42.8
	16–30 Minutes	168	35.1
	31–60 Minutes	77	16.1
	61–120 Minutes	16	3.3
	More than 120 Minutes	13	2.7
During the past month, when did you awake in the morning?	12:00–5:59 AM	169	35.3
	6–11:59 AM	267	55.7
	12–5:59 PM	39	8.1
	6–11:59 PM	4	0.8
During the past month, how many hours you sleep every night? (This may differ from the number of hours you spend in bed)	1–2:59 Hours	40	8.4
	3–4:59 Hours	69	14.4
	5–6:59 Hours	156	32.6
	7–8:59 Hours	143	29.9
	9–10:59 Hours	56	11.7
	More than 11 Hours	15	3.1

### 3.4 During the past month, how often have you had trouble sleeping?

The majority of respondents (35.7%) reported experiencing difficulty falling asleep three or more times a week, while 24.8% reported once or twice a week. The majority of respondents reported waking up in the middle of the night or early in the morning three or more times a week (46.6%), having to get up to go to the bathroom three or more times a week (48.4%), that they did not experience difficulty breathing comfortably during the past month (54.7%), that they did not experience loud coughing or snoring during the last month (50.3%). However, only around 20% respondents reported that they felt cold once or twice a week, Similarly, 21.7% felt very cold three or more times a week, 17.3% felt very hot once or twice a week, while 16.1% felt cold three or more times a week. The highest percentage of respondents (42.4%), reported not having bad dreams during last month. About 35.1% of respondents reported feeling pain three or more times a week. The majority of respondents (52.2%), indicated that they did not experience the described phenomenon during the last month (Table 4).

**Table 4:** During the past month, how often have you had trouble sleeping?

Questions	Not during the last month	Less than once a week	Once or twice a week	Three or more times a week
You cannot fall asleep within 30 min	109 (22.8%)	80 (16.7%)	119 (24.8%)	171 (35.7%)
Waking up in the middle of the night or early in the morning	74 (15.4%)	72 (15.0%)	110 (23.0%)	223 (46.6%)
I had to get up to go to the bathroom	59 (12.3%)	67 (14.0%)	121 (25.3%)	232 (48.4%)
You cannot breathe comfortably	262 (54.7%)	102 (21.3%)	63 (13.2%)	52 (10.9%)
Loud coughing or snoring	241 (50.3%)	96 (20.0%)	62 (12.9%)	80 (16.7%)
Feeling very cold	176 (36.7%)	102 (21.3%)	97 (20.3%)	104 (21.7%)
Feeling very hot	215 (44.9%)	104 (21.7%)	83 (17.3%)	77 (16.1%)
You have bad dreams	203 (42.4%)	127 (26.5%)	76 (15.9%)	73 (15.2%)
Feeling pain	168 (35.1%)	113 (23.6%)	84 (17.5%)	114 (23.8%)
Other causes	250 (52.2%)	95 (19.8%)	69 (14.4%)	65 (13.6%)

### 3.5 Over the past month, how would you rate your sleep quality in general

For the above question, the majority of respondents, 46.3%, rated their sleep quality as “Fairly Good,” followed by 38.0% who rated it as “Very Good.” A small percentage (15.7%) rated their sleep quality as “Bad” or “Very Bad.”

### 3.6 Frequency of sleep medication use and daytime sleepiness in the past month

The majority of respondents (78.5%) reported not taking any medications to help them sleep during the last month. In addition, the majority of respondents, 64.5%, reported not having trouble staying awake while driving, while eating meals, or while engaging in social activities (Table 5).

**Table 5:** Frequency of sleep medication use and daytime sleepiness in the past month.

Question	Not during the last month	Less than once a week	Once or twice a week	Three or more times a week
Over the past month, how often have you taken prescription or over-the-counter medications to help you sleep	376 (78.5%)	48 (10.0%)	26 (5.4%)	29 (6.1%)
Over the past month, how often have you had trouble staying awake while driving, while eating meals, or while engaging in social activities?	309 (64.5%)	98 (20.5%)	46 (9.6%)	26 (5.4%)

### 3.7 Do you have a bed partner or share a room

The majority of respondents, 56.36% (*n* = 270), reported having a bed partner in the same bed, while 14.2% (*n* = 68) reported a room partner but not sharing the bed, followed by 23.6% (*n* = 113) who reported not having a bed partner and room partner, and 5.8% (*n* = 28) stated they had their partner in another room. Overall, 338 (70.56%) participants were having bed and/or room partners For the 338 participants, we asked their bed and/or room partners frequency of loud snoring within the past month.

### 3.8 Snoring details

#### 3.8.1 Inquiring about the frequency of loud snoring within the past month, inquire with a bed partner or roommate (*n*-338)

The majority of participants reported not experiencing loud snoring (47.2%) or long pauses between breaths during sleep (62.6%) within the past month. Some participants reported experiencing leg restlessness during sleep (12.1%) once or twice a week, or bouts of confusion during sleep (10.9%) at least once a week. However, discomfort during sleep was reported less frequently, with only 29.3% of participants reporting such experiences less than once a week (Table 6).

**Table 6:** Frequency of loud snoring within the past month as reported by bed partners or roommates (*n* = 338).

Question	Not during the last month	Less than once a week	Once or twice a week	Three or more times a week
Loud snoring	160 (47.33%)	84 (24.85%)	43 (12.72%)	51 (15.08%)
Long pause between breaths during sleep	212 (62.72%)	83 (24.55%)	27 (7.98%)	16 (4.73%)
Leg (feet) restless during sleep	165 (48.81%)	73 (21.59%)	41 (12.13%)	59 (17.45%)
Bouts of confusion during sleep	182 (53.84%)	76 (22.48%)	37 (10.94%)	42 (12.42%)
Any discomfort during sleep	137 (40.53%)	99 (29.28%)	47 (13.90%)	55 (16.27%)

### 3.9 Pittsburgh Sleep Quality Index (PSQI) scoring and global score

The table represents the PSQI score in seven components. The Subjective Sleep Quality showed majority of respondents (53.4%) had some difficulties. Maximum respondents (55.3%) had some difficulty in Sleep Latency component. The Sleep Duration component showed that a majority of respondents (62.4%) experienced some difficulty with sleep duration. A significant majority of respondents (65.1%) reported severe difficulty in habitual sleep efficiency. The Sleep Disturbances component showed that a vast majority of respondents (85.2%) experienced some level of sleep disturbance. The majority of respondents (88.5%) reported no difficulty in sleep, while 5.4% reported some difficulty, and 6.1% reported severe difficulty in using sleep medication. For Daytime Dysfunction, a majority of respondents (48.2%) experienced some level of daytime dysfunction (Table 7).

**Table 7:** PSQI scoring and global score.

Component	No difficulty	Some difficulty	Severe difficulty
Subjective sleep quality	182 (38.0%)	256 (53.4%)	41 (8.6%)
Sleep latency	109 (22.8%)	265 (55.3%)	105 (21.9%)
Sleep duration	71 (14.8%)	299 (62.4%)	109 (22.8%)
Habitual sleep efficiency	91 (19.0%)	76 (15.9%)	312 (65.1%)
Sleep disturbances	10 (2.1%)	408 (85.2%)	61 (12.7%)
Use of sleep medication	424 (88.5%)	26 (5.4%)	29 (6.1%)
Daytime dysfunction	231 (48.2%)	222 (46.3%)	26 (5.4%)
Global PSQI Mean: 9.04 + 1.660	----	479 (100%)	----

Finally, the Global PSQI score was only available for those who completed the full questionnaire, and it showed that all participants (100%) experienced some level of sleep difficulty. This is because the component scores are summed to produce a global score (range 0 to 21). Higher scores indicate worse sleep quality and lower scores indicted good sleep quality. The mean sleep score recorded as Mean: 9.04 + 1.660.

### 3.10 Chi-square test of significance among different variables

The chi-square test is a statistical method used to determine if there’s a significant association between categorical variables. The chi-square test of significance was conducted to determine if there was a significant association between demographics, clinical characteristics and different sleep characteristics:

#### 3.10.1 Subjective sleep quality

The results showed that no significant association was found among all demographics and subjective sleep quality. This suggests that there is no significant relationship between the demographics and Subjective Sleep Quality (Table 8).

**Table 8:** Chi square test of significance among subjective sleep quality.

Demographics		No difficulty	Some difficulty	Severe difficulty	*p*-value
Age group	18–37 Years	49	78	5	0.192
	38–57 years	94	128	23	
	58–77 years	37	46	13	
	78–97 years	2	4	0	
Gender	Male	110	144	30	0.113
	Female	72	112	11	
Marital status	Bachelor	28	36	1	0.256
	Divorced/widowed	13	22	3	
	Married	141	198	37	
Education	Basic education (intermediate primary or secondary education)	70	108	13	0.374
	Illiterate (cannot read or write)	18	35	5	
	Postgraduate	10	19	2	
	University education (diploma or bachelor’s degree)	84	94	21	
Region	Central and Eastern Region (Riyadh and Eastern Regions)	39	60	11	0.642
	Southern region (Aseer, Al-Baha, Najran and Jazan regions)	68	109	19	
	The northern region (Qassim, Al-Jawf, Hail, Tabuk and the northern border regions)	27	29	4	
	Western region (Makkah and Madinah regions)	48	58	7	
Nationality	Non-Saudi	7	2	1	0.086
	Saudi	175	254	40	
Income	I have no income and depend on others to spend	45	63	5	0.677
	Less than 5,000 Saudi riyals	40	63	10	
	More than 15,000 Saudi riyals	33	44	8	
	More than 5,000–15,000 Saudi riyals	64	86	18	
Duration of diabetes	less than 5 year	57	78	12	0.997
	From 5 to 10 years	28	42	7	
	More than 10 Years	97	136	22	
Glycosylated hemoglobin test (HBA1C)	Less than 7%	56	81	16	0.514
	From 7 to less than 9%	84	107	14	
	From 9 to less than 11%	24	35	7	
	More than 11%	14	19	1	
	I don’t know	4	14	3	
Comorbidity	Anxiety	10	4	0	0.073
	Cardiovascular disease	2	8	2	
	Depression	2	6	1	
	Diabetes	76	99	22	
	Hypertension	44	82	9	
	Dyslipidemia	48	57	7	

#### 3.10.2 Sleep latency

The chi-square test results showed that except nationality (*p* = 0.01), no significant association was found among remaining demographics and sleep latency (Supplementary Table S1).

#### 3.10.3 Sleep duration

The chi-square test results showed that except nationality (*p* = 0.006) no significant association was found among remaining demographics and sleep duration (Supplementary Table S2).

#### 3.10.4 Habitual sleep efficiency

The chi-square test results showed that marital status (*p* = 0.022), nationality (*p* = 0.006), Glycosylated hemoglobin test (HBA1C) (*p* = 0.044) and Comorbidity (*p* < 0.001) had significant association with Habitual Sleep Efficiency while no significant association was found among remaining demographics (Supplementary Table S3).

#### 3.10.5 Sleep disturbances

The chi-square test results showed that education (*p* = 0.001) and nationality (*p* = 0.031) had significant association with sleep disturbances while, no significant association was found among remaining demographics (Supplementary Table S4).

#### 3.10.6 Use of sleep medication

The chi-square test results showed that gender (*p* = 0.040), region (*p* = 0.031) and nationality (*p* = 0.002) had significant association with use of sleep medication while, no significant association was found among remaining demographics (Supplementary Table S5).

#### 3.10.7 Daytime dysfunction

The chi-square test results showed that gender (*p* = 0.023), marital status (*p* = 0.012), education (*p* = 0.037), income (*p* = 0.027), Glycosylated hemoglobin test (HBA1C) (*p* = 0.028) and comorbidity (*p* = 0.020) had significant association with daytime dysfunction (Supplementary Table S6).

### 3.11 Factors affecting PSQI global score: a regression analysis

The table displays the unstandardized coefficients, standardized coefficients, *t*-values, significance level, and confidence intervals for the independent variables in predicting the dependent variable Global PSQI. The results show that the variables age group, gender, marital status, region, education, nationality, income, duration, last blood Hba1c, and comorbidity all have coefficients that contribute to predicting Global PSQI. Among these variables, education has the largest negative standardized coefficient (−0.113), indicating that lower levels of education are associated with higher scores on the Global PSQI. Comorbidity also has a significant positive coefficient (0.106), suggesting that having comorbidities is associated with higher scores on the Global PSQI. The other variables have smaller coefficients that are not statistically significant at the conventional 0.05 level. Overall, the results suggest that education and comorbidity are the most important factors in predicting Global PSQI (Table 9).

**Table 9:** Factors affecting PSQI global score: a regression analysis.

Model	Unstandardized coefficients		Standardized coefficients	*t*	Sig. (*p*-value)	95.0% Confidence interval for B	
	*B*	Std. error	Beta			Lower bound	Upper bound
(Constant)	8.403	1.116	–	7.529	0.000	6.210	10.596
Age group	0.141	0.114	0.062	1.238	0.216	−0.083	0.366
Gender numeric	0.335	0.180	0.099	1.862	0.063	−0.018	0.688
Marital numeric	0.000	0.123	0.000	−0.004	0.997	−0.242	0.241
Region numeric	−0.018	0.070	−0.012	−0.259	0.796	−0.156	0.120
Education numeric	−0.183	0.076	−0.113	−2.409	0.016*	−0.333	−0.034
Nationality	0.377	0.533	0.032	0.706	0.480	−0.671	1.425
Income (monthly)	−0.032	0.081	−0.023	−0.398	0.690	−0.191	0.127
Duration	0.130	0.085	0.069	1.518	0.130	−0.038	0.297
Last blood Hba1c	−0.050	0.071	−0.032	−0.710	0.478	−0.190	0.089
Comorbidity	0.157	0.068	0.106	2.320	0.021*	0.024	0.290

*p < 0.05.

## 4 Discussion

The study findings indicated that there is a notable prevalence of sleep problems among patients diagnosed with type 2 diabetes mellitus (T2DM) in Saudi Arabia. The majority of responders stated difficulty with sleep duration, habitual sleep efficiency, and sleep disruption. Additionally, a considerable majority of subjects reported feeling some level of daytime dysfunction. Healthcare practitioners should consider the examination and treatment of sleep disturbances among individuals with T2DM as part of comprehensive care, while also addressing other life-threatening and vision-threatening issues. Healthcare practitioners could consider including routine screening for sleep disturbances as part of the usual care for individuals with T2DM. Furthermore, addressing the underlying causes of sleep problems, including comorbid diseases, may assist in improving overall health outcomes for people with T2DM. Numerous studies have reported that inadequate sleep quality is a potential risk factor for diabetes (4, 19–21). The result was in line with other research demonstrating a link between PSQ and both diabetes mellitus and hypertension (22, 23).

The findings of the study revealed that the majority of participants reported going to bed between 12:00 AM and 5:59 AM and waking up between 6 AM and 11:59 AM, indicating their sleep patterns within these time frames. Furthermore, the majority of participants reported a sleep onset latency of 1–15 min and a total sleep time of 5–6.59 h per night. The results indicate that people diagnosed with diabetes may experience disturbed sleep cycles, which could potentially play a role in the onset and treatment of their ailment. Consequently, these findings may hold significant ramifications for sleep health education and interventions aimed at individuals with diabetes. Healthcare providers can potentially enhance the management of diabetes and improve overall health outcomes by imparting knowledge on the significance of healthy sleep habits and implementing interventions that enhance sleep duration and quality (24).

This research offers significant contributions to the understanding of the incidence and pervasiveness of diverse sleep disruptions and indications encountered by persons diagnosed with diabetes. The results indicate that a considerable proportion of respondents experienced challenges in initiating sleep and maintaining it, either by waking up in the middle of the night or early in the morning. Such disturbances can have a substantial effect on their overall sleep efficacy. Furthermore, a significant proportion of the respondents indicated experiencing sensations of cold or heat, discomfort, and frequent nocturia every week. The study additionally underscores that a subset of participants indicated an absence of negative dreams, thereby constituting a favorable outcome. The above-mentioned observations could prove valuable for healthcare practitioners in detecting potential sleep-related issues among individuals with diabetes and devising focused interventions to enhance their sleep quality and general health consequences (25).

Another finding of the current study stated that while most participants perceive their sleep quality to be good, there is a significant minority that is experiencing poor sleep quality. These insights may be useful for healthcare providers in identifying individuals with diabetes who may be experiencing poor sleep quality and developing targeted interventions to improve their sleep health and overall well-being. The results were in line with previous research indicating that individuals with diabetes mellitus frequently experience suboptimal sleep quality (26). The prevalence of poor sleep quality among individuals with diabetes mellitus was found to be 47.6% on a global scale (27). Sleep disturbance was observed in 29.5% of the diabetic population in Africa (28). According to a study conducted in Ethiopia, sleep disturbance was found to affect approximately 55.6% of individuals diagnosed with diabetes (4).

The research offers significant insights into the utilization of medication and the prevalence of daytime sleepiness among the subjects under investigation. The results indicate that a majority of the respondents refrained from taking any medication in the preceding month, and a considerable proportion did not report experiencing any difficulty in maintaining alertness during their daily activities. The findings indicate that the influence of medication consumption and daytime sleepiness on the suboptimal quality of sleep in this cohort may not be substantial. Nevertheless, additional investigation is required to examine other plausible variables that could be influencing the quality of sleep among individuals with diabetes. The said observations could potentially serve as valuable guidance for healthcare practitioners in devising focused measures aimed at enhancing the sleep health of this demographic (24).

The current study demonstrated that the Global Pittsburgh Sleep Quality Index (PSQI) score indicated that all of the participants (*n* = 479; 100%) experienced some degree of sleep disturbance. This is because the component scores are summed to produce a global score (range 0 to 21). Higher scores indicate worse sleep quality and lower scores indicted good sleep quality. The mean sleep score recorded as Mean: 9.04 + 1.660. The findings are consistent, 41.1% of type II diabetic patients had poor sleep quality, as measured by the PSQI, a subjective tool with a cut-off of 5. While utilizing a cut-off level of 8 for the PSQI, a different study of a similar nature was conducted in Singapore, where the rate of poor sleep quality was 47.1% (29). In Brazil, 52% of type II diabetic patients reported having bad sleep (30). In North Korea, 48.2% of type II diabetes patients reported having sleeplessness frequently (22).

Previous research has documented elevated prevalence rates of suboptimal sleep quality, ranging from 69 to 73.9%, when utilizing a PSQI cut-off score of 5 (6, 31). Lecube et al. (27) reported that individuals with Type 2 Diabetes Mellitus (T2DM) in Spain exhibited greater sleep disturbances in comparison to control participants. Comparing studies, including the current one, presents certain challenges due to variations in patient characteristics, particularly age, which is a recognized factor that may influence sleep quality.

The study’s results suggest that various demographic and health-related variables play a role in forecasting Global PSQI. Education and comorbidity emerge as the primary predictors of Global PSQI among the aforementioned factors. The standardized coefficient pertaining to education exhibits a negative association, indicating that higher scores on the Global PSQI are associated with lower levels of education. The aforementioned discovery underscores the potential significance of educational interventions that target the enhancement of sleep quality among individuals with comparatively lower levels of education (32). The presence of comorbidity is positively correlated with elevated scores on the Global PSQI, indicating that the co-occurrence of multiple chronic conditions is linked to increased severity of sleep disturbances. The above-mentioned finding highlights the necessity of providing all-encompassing care and effective management of concurrent medical conditions in persons experiencing sleep disruptions (33). Although statistical significance was not achieved for the other variables examined in this investigation, their coefficients could still offer valuable insights into the associations between demographic and health-related factors and the quality of sleep. In light of these results, it appears that a comprehensive strategy is required to tackle sleep disruptions among individuals with diabetes, which should consider various factors such as education and comorbidities that are related to demographics and health. The findings were consistent with other findings that showed the prevalence of prevalence of low sleep quality was observed to be considerably greater among female patients in comparison to their male counterparts, and among individuals aged 60 years or above as opposed to those in younger age groups. This finding is consistent with a prior investigation that demonstrated inferior sleep quality in females and older adults (34).

Regular exercise has been shown to have positive effects on sleep quality and duration in the general population, and similar benefits have been observed in individuals with diabetes. In a recent study conducted by Amiri et al. (35), researchers investigated the impact of physical exercise on the sleep patterns of children diagnosed with type 1 diabetes. The study revealed that the sleep patterns of the participants in the experimental group showed significant improvement following an 8-week exercise training program that incorporated educational videos (35). In another study, Fritz et al. (36), reported that the quality of sleep improved in the group with normal glucose tolerance following 4 months of Nordic walking.

Multiple studies have provided evidence that exercise has the potential to enhance the quality of sleep, alleviate symptoms of depression, and serve as a preventive measure against chronic diseases (37–39). Thus, incorporating exercise into the treatment plan can be a beneficial approach for managing diabetes and enhancing sleep quality in individuals with diabetes.

## 5 Conclusion

To summarize, the research offers novel perspectives on the determinants that impact the Global Pittsburgh Sleep Quality Index (PSQI) among individuals diagnosed with diabetes. The findings underscore the importance of educational programs and comprehensive interventions that take into account the presence of comorbidities in order to improve the quality of sleep. Prior research has indicated that suboptimal sleep quality is more prevalent among females and older individuals, a finding that is corroborated by the present study. The findings of this study may serve as a basis for developing efficacious interventions targeted toward improving the well-being and overall health of individuals afflicted with diabetes. Further investigation is necessary to comprehensively comprehend the impacts of these therapies and to precisely identify additional plausible factors contributing to suboptimal sleep quality within this particular group.

## 6 Study limitations

The study’s generalizability may be impacted by limitations related to the sample size, which is a potential concern. In the event of a small sample size, the representation of the heterogeneous population of individuals diagnosed with diabetes may be insufficient, thereby constraining the external validity of the findings. The study recognizes that inadequate sleep quality is a more widespread issue among women and individuals of advanced age. It is possible that the characteristics of the sample may not be entirely representative of the wider population of individuals who have diabetes. The potential consequence of this is a restricted applicability of the results to alternative demographic cohorts.

The study referred to in the conclusion employs a cross-sectional design, which entails the collection of data at a particular moment in time. The use of a cross-sectional design in this study imposes constraints on the ability to establish causal relationships between the determinants and sleep quality, as the design inherently restricts causal inferences. Conducting longitudinal studies would yield more reliable and comprehensive evidence regarding the various factors that impact the quality of sleep over an extended period. The research presumably utilized self-report measures to evaluate sleep quality and other variables. Self-report measures are prone to subjectivity and may be susceptible to recall bias or social desirability bias. The utilization of objective measures, such as polysomnography or actigraphy, would yield more precise and dependable information pertaining to the quality of sleep.

The study’s deficiency in having a control group impedes the capacity to contrast the sleep quality of individuals with diabetes to a population without diabetes. Incorporating a control group into the research design would yield significant contributions toward elucidating the precise effects of diabetes on the quality of sleep. The study acknowledges the existence of confounding factors, specifically comorbidities that have an impact on the quality of sleep. Nonetheless, it is plausible that there exist additional confounding factors that were not considered during the course of the analysis. The presence of unmeasured or unidentified variables may have the potential to affect the association between determinants and the quality of sleep.

One potential limitation of this study is that it does not comprehensively investigate all possible risk factors associated with sleep disturbance among individuals with type 2 diabetes. Factors such as obesity, smoking, and other lifestyle variables known to influence sleep patterns were not included in the scope of this research. Consequently, the study’s findings may not provide a complete understanding of the complex interplay between sleep disturbance and these important risk factors. Future studies should consider incorporating a broader range of variables to capture a more comprehensive picture of the factors contributing to sleep disturbances in this population.

The issue of generalizability arises as the research may have been carried out in a particular healthcare setting or geographic region. The generalizability of the results may be limited as the applicability of the findings to individuals with diabetes from diverse cultural backgrounds or healthcare systems is uncertain.

## 7 Recommendations

Developing interventions based on the findings of the study can indeed be helpful in addressing sleep problems in individuals with diabetes. Tailoring interventions to specific predictors can improve their effectiveness. Here are some potential intervention strategies that could be considered based on the significant predictors identified in the study:

### 7.1 Marital status

For individuals who are married or in a committed relationship, involving their partners in the intervention may be beneficial. Couples-focused interventions can address issues such as sleep disruptions caused by snoring, bed-sharing conflicts, or different sleep schedules. Providing education and communication strategies to improve sleep hygiene for both partners can contribute to better sleep quality.

### 7.2 Nationality and region

Cultural factors can influence sleep habits and preferences. Developing culturally sensitive interventions that consider specific cultural practices, beliefs, and norms related to sleep may be helpful. These interventions can provide education about healthy sleep practices while respecting cultural differences. Additionally, providing information in the participants’ native language can enhance understanding and engagement.

### 7.3 HBA1C (glycemic control)

Improving diabetes management and glycemic control can positively impact sleep quality. Interventions can focus on optimizing diabetes treatment plans, including medication adherence, dietary modifications, and regular physical activity. Collaborating with healthcare professionals, such as endocrinologists or certified diabetes educators, can ensure that sleep-related issues are considered in the overall diabetes care plan.

### 7.4 Education

Educating participants about the relationship between diabetes and sleep problems can raise awareness and motivate behavior change. Interventions can provide information about the impact of sleep disturbances on diabetes management and overall health. This education can be delivered through workshops, informational materials, or digital platforms that offer personalized content based on participants’ educational backgrounds.

### 7.5 Comorbidity

Addressing comorbid conditions that are associated with sleep disturbances, such as depression or anxiety, is essential. Integrating mental health support into the intervention can involve psychoeducation, cognitive-behavioral therapy techniques, or referral to mental health professionals for further evaluation and treatment.

### 7.6 Monthly income

Financial constraints may limit access to resources that promote better sleep, such as comfortable bedding or noise-canceling devices. Interventions can provide information on low-cost or free resources available in the community, such as sleep clinics, support groups, or relaxation techniques that can be practiced at home.

It is important to note that these are general suggestions, and the specific design and implementation of interventions should be based on further research, feasibility, and the needs of the target population. Tailoring interventions to individual predictors can improve their relevance and effectiveness in addressing sleep problems in individuals with diabetes. In order to enhance the sleep outcomes of people with diabetes, a multifaceted approach that targets the many components affecting sleep quality is required.

## Data availability statement

The original contributions presented in the study are included in the article/Supplementary material, further inquiries can be directed to the corresponding author.

## Ethics statement

The studies involving humans were approved by the Research Ethics Committee at King Khalid University, Abha, Saudi Arabia. The studies were conducted in accordance with the local legislation and institutional requirements. The participants provided their written informed consent to participate in this study.

## Author contributions

FA-Q: Writing – original draft, Methodology, Funding acquisition, Conceptualization.

## Footnotes

^1^ http://www.raosoft.com/samplesize.html

## Supplementary material

Supplementary File 1 Chi square test results of significance among different study variables.

Supplementary File 2 Study questionnaire in Arabic language.

Supplementary File 3 Study questionnaire in English language.
