# Supplementary material for: Prevalence of sleep disturbance and its associated factors among diabetes type-2 patients in Saudi Arabia
Source: Front Public Health. 2024 Nov 1;12:1283629. doi: 10.3389/fpubh.2024.1283629 (PMC11563833; doi:10.3389/fpubh.2024.1283629)
Supplement: Supplementary File 1 — Chi square test results of significance among different study variables. [file Data_Sheet_1.docx]

**Prevalence of Sleep Disturbance and its Associated Factors among People Living With Type 2 Diabetes in Saudi Arabia**

**Supplementary File**

**Table S1. Chi square test of significance among Sleep Latency**

| **Demographics** | | **No difficulty** | **Some Difficulty** | **Severe Difficulty** | **P value** |
| --- | --- | --- | --- | --- | --- |
| **Age Group** | 18-37 Years  38-57 years  58-77 years  78-97 years | 31  59  17  2 | 71  135  57  2 | 30  51  22  2 | 0.805 |
| **Gender** | Male  Female | 66  43 | 102  163 | 50  55 | 0.261 |
| **Marital Status** | Bachelor  Divorced/widowed  Married | 14  6  89 | 30  21  214 | 21  11  73 | 0.122 |
| **Education** | Basic education (intermediate primary or secondary education)  Illiterate (cannot read or write)  Postgraduate  University education (diploma or bachelor's degree) | 43  12  11  43 | 110  32  16  107 | 38  14  4  49 | 0.534 |
| **Region** | Central and Eastern Region (Riyadh and Eastern Regions)  Southern region (Aseer, Al-Baha, Najran and Jazan regions)  The northern region (Qassim, Al-Jawf, Hail, Tabuk and the northern border regions)  Western region (Makkah and Madinah regions) | 26  45  15  23 | 57  112  31  65 | 27  39  14  25 | 0.934 |
| **Nationality** | Non-Saudi  Saudi | 2  107 | 2  263 | 6  99 | **0.01** |
| **Income** | I have no income and depend on others to spend  Less than 5000 Saudi riyals  More than 15,000 Saudi riyals  More than 5,000 to 15,000 Saudi riyals | 23  30  21  35 | 60  54  48  103 | 30  29  16  30 | 0.298 |
| **Duration of diabetes** | less than 5 year  From 5 to 10 years  More than 10 Years | 37  21  51 | 81  39  145 | 29  17  59 | 0.587 |
| **Glycosylated hemoglobin test (HBA1C)** | Less than 7%  From 7 to less than 9%  From 9 to less than 11%  More than 11%  I Dont Know | 38  43  10  11  7 | 87  117  39  11  11 | 28  45  17  12  3 | 0.113 |
| **Comorbidity** | Anxiety  Cardiovascular disease  Depression  Diabetes  Hypertension  Dyslipidemia | 2  4  3  44  31  25 | 7  7  4  115  79  53 | 5  1  2  38  25  34 | 0.369 |

**Table S2. Chi square test of significance among Sleep Duration**

| **Demographics** | | **No difficulty** | **Some Difficulty** | **Severe Difficulty** | **P value** |
| --- | --- | --- | --- | --- | --- |
| **Age Group** | 18-37 Years  38-57 years  58-77 years  78-97 years | 21  30  19  1 | 79  157  60  3 | 32  58  17  2 | 0.570 |
| **Gender** | Male  Female | 35  36 | 184  115 | 65  44 | 0.168 |
| **Marital Status** | Bachelor  Divorced/widowed  Married | 13  9  49 | 37  23  239 | 15  6  88 | 0.258 |
| **Education** | Basic education (intermediate primary or secondary education)  Illiterate (cannot read or write)  Postgraduate  University education (diploma or bachelor's degree) | 25  12  3  31 | 122  34  17  126 | 44  12  11  42 | 0.489 |
| **Region** | Central and Eastern Region (Riyadh and Eastern Regions)  Southern region (Aseer, Al-Baha, Najran and Jazan regions)  The northern region (Qassim, Al-Jawf, Hail, Tabuk and the northern border regions)  Western region (Makkah and Madinah regions) | 14  26  9  22 | 68  126  36  69 | 28  44  15  22 | 0.740 |
| **Nationality** | Non-Saudi  Saudi | 5  66 | 3  296 | 2  107 | **0.006** |
| **Income** | I have no income and depend on others to spend  Less than 5000 Saudi riyals  More than 15,000 Saudi riyals  More than 5,000 to 15,000 Saudi riyals | 21  19  12  19 | 67  64  54  114 | 25  30  19  35 | 0.492 |
| **Duration of diabetes** | less than 5 year  From 5 to 10 years  More than 10 Years | 21  10  40 | 89  48  162 | 37  19  53 | 0.844 |
| **Glycosylated hemoglobin test (HBA1C)** | Less than 7%  From 7 to less than 9%  From 9 to less than 11%  More than 11%  I Dont Know | 19  30  11  8  3 | 97  132  44  15  11 | 37  43  11  11  7 | 0.350 |
| **Comorbidity** | Anxiety  Cardiovascular disease  Depression  Diabetes  Hypertension  Dyslipidemia | 1  0  1  24  20  25 | 11  8  5  131  83  61 | 2  4  3  42  32  26 | 0.307 |

**Table S3. Chi square test of significance among Habitual Sleep Efficiency**

| **Demographics** | | **No difficulty** | **Some Difficulty** | **Severe Difficulty** | **P value** |
| --- | --- | --- | --- | --- | --- |
| **Age Group** | 18-37 Years  38-57 years  58-77 years  78-97 years | 27  43  20  1 | 20  42  13  1 | 85  160  63  4 | 0.975 |
| **Gender** | Male  Female | 44  47 | 55  21 | 185  127 | 0.007 |
| **Marital Status** | Bachelor  Divorced/widowed  Married | 19  11  61 | 8  2  66 | 38  25  249 | **0.022** |
| **Education** | Basic education (intermediate primary or secondary education)  Illiterate (cannot read or write)  Postgraduate  University education (diploma or bachelor's degree) | 32  13  4  42 | 33  4  4  35 | 126  41  23  122 | 0.341 |
| **Region** | Central and Eastern Region (Riyadh and Eastern Regions)  Southern region (Aseer, Al-Baha, Najran and Jazan regions)  The northern region (Qassim, Al-Jawf, Hail, Tabuk and the northern border regions)  Western region (Makkah and Madinah regions) | 21  35  12  23 | 15  26  14  21 | 74  135  34  69 | 0.477 |
| **Nationality** | Non-Saudi  Saudi | 6  85 | 1  75 | 3  309 | **0.004** |
| **Income** | I have no income and depend on others to spend  Less than 5000 Saudi riyals  More than 15,000 Saudi riyals  More than 5,000 to 15,000 Saudi riyals | 26  26  15  24 | 17  12  15  32 | 70  75  55  112 | 0.279 |
| **Duration of Diabetes** | less than 5 year  From 5 to 10 years  More than 10 Years | 24  14  53 | 28  14  34 | 95  49  168 | 0.510 |
| **Glycosylated hemoglobin test (HBA1C)** | Less than 7%  From 7 to less than 9%  From 9 to less than 11%  More than 11%  I Dont Know | 22  39  15  12  3 | 29  36  9  2  0 | 102  130  42  20  18 | **0.044** |
| **Comorbidity** | Anxiety  Cardiovascular disease  Depression  Diabetes  Hypertension  Dyslipidemia | 2  0  2  30  24  33 | 8  2  0  37  17  12 | 4  10  7  130  94  67 | **0.001** |

**Table S4. Chi square test of significance among Sleep Disturbances**

| **Demographics** | | **No difficulty** | **Some Difficulty** | **Severe Difficulty** | **P value** |
| --- | --- | --- | --- | --- | --- |
| **Age Group** | 18-37 Years  38-57 years  58-77 years  78-97 years | 3  4  3  0 | 112  214  77  5 | 17  27  16  1 | 0.795 |
| **Gender** | Male  Female | 7  3 | 247  161 | 30  31 | 0.190 |
| **Marital Status** | Bachelor  Divorced/widowed  Married | 2  1  7 | 53  29  326 | 10  8  43 | 0.416 |
| **Education** | Basic education (intermediate primary or secondary education)  Illiterate (cannot read or write)  Postgraduate  University education (diploma or bachelor's degree) | 4  1  4  1 | 163  46  25  174 | 24  11  2  24 | **0.001** |
| **Region** | Central and Eastern Region (Riyadh and Eastern Regions)  Southern region (Aseer, Al-Baha, Najran and Jazan regions)  The northern region (Qassim, Al-Jawf, Hail, Tabuk and the northern border regions)  Western region (Makkah and Madinah regions) | 5  2  1  2 | 91  174  52  91 | 14  20  7  20 | 0.220 |
| **Nationality** | Non-Saudi  Saudi | 0  10 | 6  402 | 4  57 | **0.031** |
| **Income** | I have no income and depend on others to spend  Less than 5000 Saudi riyals  More than 15,000 Saudi riyals  More than 5,000 to 15,000 Saudi riyals | 2  3  4  1 | 91  94  74  149 | 20  16  7  18 | 0.138 |
| **Duration of Diabetes** | less than 5 year  From 5 to 10 years  More than 10 Years | 4  2  4 | 124  68  216 | 19  7  35 | 0.769 |
| **Glycosylated hemoglobin test (HBA1C)** | Less than 7%  From 7 to less than 9%  From 9 to less than 11%  More than 11%  I Don’t Know | 4  3  1  2  0 | 132  176  54  27  19 | 17  26  11  5  2 | 0.737 |
| **Comorbidity** | Anxiety  Cardiovascular disease  Depression  Diabetes  Hypertension  Dyslipidemia | 0  0  0  1  4  5 | 13  12  9  177  112  85 | 1  0  0  19  19  22 | 0.058 |

**Table S5. Chi-square test of significance among Use of Sleep Medication**

| **Demographics** | | **No difficulty** | **Some Difficulty** | **Severe Difficulty** | **P value** |
| --- | --- | --- | --- | --- | --- |
| **Age Group** | 18-37 Years  38-57 years  58-77 years  78-97 years | 118  220  81  5 | 8  12  5  1 | 6  13  10  0 | 0.424 |
| **Gender** | Male  Female | 260  164 | 12  14 | 12  17 | **0.040** |
| **Marital Status** | Bachelor  Divorced/widowed  Married | 57  31  336 | 4  2  20 | 4  5  20 | 0.431 |
| **Education** | Basic education (intermediate primary or secondary education)  Illiterate (cannot read or write)  Postgraduate  University education (diploma or bachelor's degree) | 169  47  30  178 | 9  3  1  13 | 13  8  0  8 | 0.098 |
| **Region** | Central and Eastern Region (Riyadh and Eastern Regions)  Southern region (Aseer, Al-Baha, Najran and Jazan regions)  The northern region (Qassim, Al-Jawf, Hail, Tabuk and the northern border regions)  Western region (Makkah and Madinah regions) | 97  179  54  94 | 2  8  4  12 | 11  9  2  7 | **0.031** |
| **Nationality** | Non-Saudi  Saudi | 6  418 | 3  23 | 1  28 | **0.002** |
| **Income** | I have no income and depend on others to spend  Less than 5000 Saudi riyals  More than 15,000 Saudi riyals  More than 5,000 to 15,000 Saudi riyals | 95  98  80  151 | 7  7  3  9 | 11  8  2  8 | 0.364 |
| **Duration of Diabetes** | less than 5 year  From 5 to 10 years  More than 10 Years | 130  70  224 | 10  2  14 | 7  5  17 | 0.681 |
| **Glycosylated hemoglobin test (HBA1C)** | Less than 7%  From 7 to less than 9%  From 9 to less than 11%  More than 11%  I Don’t Know | 136  182  57  29  20 | 12  9  3  2  0 | 5  14  6  3  1 | 0.521 |
| **Comorbidity** | Anxiety  Cardiovascular disease  Depression  Diabetes  Hypertension  Dyslipidemia | 13  12  9  180  118  92 | 1  0  0  12  6  7 | 0  0  0  5  11  13 | 0.111 |

**Table S6. Chi square test of significance among Daytime Dysfunction**

| **Demographics** | | **No difficulty** | **Some Difficulty** | **Severe Difficulty** | **P value** |
| --- | --- | --- | --- | --- | --- |
| **Age Group** | 18-37 Years  38-57 years  58-77 years  78-97 years | 53  125  50  3 | 73  108  38  3 | 6  12  8  0 | 0.228 |
| **Gender** | Male  Female | 150  81 | 123  99 | 11  15 | **0.023** |
| **Marital Status** | Bachelor  Divorced/widowed  Married | 21  18  192 | 40  15  167 | 4  5  17 | **0.012** |
| **Education** | Basic education (intermediate primary or secondary education)  Illiterate (cannot read or write)  Postgraduate  University education (diploma or bachelor's degree) | 96  29  12  94 | 82  22  19  99 | 13  7  0  6 | **0.037** |
| **Region** | Central and Eastern Region (Riyadh and Eastern Regions)  Southern region (Aseer, Al-Baha, Najran and Jazan regions)  The northern region (Qassim, Al-Jawf, Hail, Tabuk and the northern border regions)  Western region (Makkah and Madinah regions) | 55  93  27  56 | 45  94  31  52 | 10  9  2  5 | 0.529 |
| **Nationality** | Non-Saudi  Saudi | 2  229 | 7  215 | 1  25 | 0.191 |
| **Income** | I have no income and depend on others to spend  Less than 5000 Saudi riyals  More than 15,000 Saudi riyals  More than 5,000 to 15,000 Saudi riyals | 49  46  39  97 | 54  59  44  65 | 10  8  2  6 | **0.027** |
| **Duration of Diabetes** | less than 5 year  From 5 to 10 years  More than 10 Years | 73  32  126 | 68  40  114 | 6  5  15 | 0.687 |
| **Glycosylated hemoglobin test (HBA1C)** | Less than 7%  From 7 to less than 9%  From 9 to less than 11%  More than 11%  I Don’t Know | 77  107  33  8  6 | 72  85  28  23  14 | 4  13  5  3  1 | **0.028** |
| **Comorbidity** | Anxiety  Cardiovascular disease  Depression  Diabetes  Hypertension  Dyslipidemia | 8  7  2  107  64  43 | 6  5  7  86  61  57 | 0  0  0  4  10  12 | **0.020** |
