# Supplementary material for: Prevalence of sleep disturbance and its associated factors among diabetes type-2 patients in Saudi Arabia
Source: Front Public Health. 2024 Nov 1;12:1283629. doi: 10.3389/fpubh.2024.1283629 (PMC11563833; doi:10.3389/fpubh.2024.1283629)
Supplement: Supplementary File 2 — Study questionnaire in Arabic language. [file Data_Sheet_2.pdf]

تعداد السكان ومعلوماتهم

العمر

______________________________ :

الجنس

ذكر ☐

أنثى ☐

الحالة الزوجية

أعزب ☐

متزوج ☐

مطلق/أرمل ☐

المنطقة

المنطقة الوسطى ☐

المنطقة الشمالية ☐

المنطقة الشرقية ☐

المنطقة الغربية ☐

المنطقة الجنوبية ☐

المستوى التعليمي

أمي ☐

أقل من الثانوية ☐

ثانوية ☐

جامعي ☐

الجنسية

سعودي ☐

غير سعودي ☐

الدخل الشهري

أقل من 5000 ريال سعودي ☐

ريال سعودي 5000-10000 ☐

أكثر من 10000 ريال سعودي ☐

مدة تشخيص السكري:

أقل من سنة ☐

سنوات 1-5 ☐

سنوات 6-10 ☐

أكثر من 10 سنوات ☐

نوع السكري:

النوع 1 ☐

النوع 2 ☐

السكري الحملي ☐

الحالات الطبية الأخرى (قم بوضع علامة في جميع الخيارات المناسبة):

ارتفاع ضغط الدم ☐

زيادة الكوليسترول في الدم ☐

أمراض القلب ☐

الاكتئاب ☐

القلق ☐

غير ذلك ☐

Pittsburgh Sleep Quality Index

مؤشر جودة النوم

التعليمات

الاسئله التاليه تتعلق بعادات نومك خلال الشهر الماضي فقط. يجب ان تشير اجاباتك بدقه الى معظم الايام والليالي في الشهر الماضي. من فضلك اجب على جميع الاسئله.

1) خلال الشهر الماضي متى كنت تذهب عادة الى الفراش ليلا؟

ميعاد النوم المعتاد .... (مثلا: 10:30 مساء)

2) خلال الشهر الماضي كم كان عدد الدقائق التي تستغرقها حتى تخلد للنوم كل ليله عادة؟

عدد الدقائق .... (مثلا 10 دقائق)

3) خلال الشهر الماضي متى كنت تنهض من الفراش في الصباح؟

ميعاد النهوض من الفراش .... (مثلا: 7:30 صباحا)

4) خلال الشهر الماضي كم كان عدد الساعات الفعليه التي تنامها كل ليله ؟ (هذا قد يختلف عن عدد الساعات التي تقضيها في الفراش)

عدد ساعات النوم كل ليله .... (مثلا: 10:30 ساعات)

اختار الاجابه الافضل لكل من الاسئله التاليه. من قضلك اجب على جميع الاسئله.

5) خلال الشهر الماضي كم مره حدثت لك مشاكل خلال النوم لانك ....

	ليس خلال الشهر الماضي	اقل من واحده قي الاسبوع	مره او مرتين في الاسبوع	ثلاث مرات او اكثر في الاسبوع
**ا) لا تستطيع النوم خلال 30 دقيقه**				
**ب) الاستيقاظ في منتصف الليل او في الصباح الباكر**				
**ج) اضطررت للاستيقاظ من اجل الذهاب الى الحمام**				
**د) لا تسطيع التنفس بارتياح**				
**ه) السعال او الشخير العالي**				
**و) الشعور بالبرد الشديد**				

ز) الشعوربالحر الشديد				
ح) تساورك احلام سيئه				
ط) الشعور بالالم				
ي) اسباب اخرى رجاء اشرح .... خلال الشهر الماضي كم مره حدثت لك مشاكل خلال النوم بسبب ذلك				

٦) خلال الشهر الماضي، كيف تقيم جودة نومك عموما

جيد جدا ----------

جيد الى حد ما----

سئ جدا-----------

سئ الى حد ما-----

	ليس خلال الشهر الماضي	اقل من واحده في الاسبوع	مره او مرتين في الاسبوع	ثلاث مرات او اكثر في الاسبوع
**7. خلال الشهر الماضي كم مره اخذت ادويه موصوفه او غير موصوفه لتساعدك على النوم**				
**8. خلال الشهر الماضي كم مره واجهت مشكله بالبقاء مستيقظا اثناء القياده او اثناء تناول وجبات الطعام او خلال الانخراط في الانشطه الاجتماعيه**				

٩) خلال الشهر الماضي، كم كان حجم المشكله لديك للحفاظ على ما يكفي من الحماسه لانجاز الامور

لا مشكله على الاطلاق--------

فقط مشكله بسيطه جدا--------

مشكلة الى حد ما-------------

مشكله كبيره جدا-------------

١٠) هل لديك شريك في الفراش او تشارك الغرفه

لا يوجد شريك في الفراش او لا تشارك الغرفه -------
شريك في غرفة اخرى -------
شريك في الغرفه وليس الفراش -------
شريك في الفراش -------

١١) اذا كان لديك شريك في الفراش او تشارك الغرفه اساله/ او اسالها خلال الشهر الماضي، كم مره كان لديك

	ليس خلال الشهر الماضي	اقل من واحده قي الاسبوع	مره او مرتين في الاسبوع	ثلاث مرات او اكثر في الاسبوع
ا) شخير بصوت عالي				
ب) وقفه طويله بيت الانفاس اثناء النوم				
ج) رجل غير هادئه اثناء النوم				
د) نوبات من الارتباك اثناء النوم				
ه) اي عدم راحه اثناء النوم: اشرح من فضلك				
