# Supplementary material for: Prevalence of sleep disturbance and its associated factors among diabetes type-2 patients in Saudi Arabia
Source: Front Public Health. 2024 Nov 1;12:1283629. doi: 10.3389/fpubh.2024.1283629 (PMC11563833; doi:10.3389/fpubh.2024.1283629)
Supplement: Supplementary File 3 — Study questionnaire in English language. [file Data_Sheet_3.pdf]

Demographic Information:

Age: ___________________________

Gender:
Male
Female

Marital Status:
Single
Married
Divorced
Widowed

Education Level:
- Below High School
- High School
- University

Diabetes Diagnosis Duration:
- Less than 1 year
- 1-5 years
- More than 5 years

Diabetes Type:
- Type 1
- Type 2
- Gestational Diabetes
- Other medical conditions (please specify)

Medical Conditions (Check all applicable):
- High blood pressure
- High cholesterol
- Heart disease
- Obesity
- Sleep disorders
- Kidney problems

Pittsburgh Sleep Quality Index:

The following questions are about your sleep habits during the past month. Please answer based on most nights in the past month.

1. **When did you usually go to bed at night?**
   Usual bedtime: __________________ (e.g., 10:30 PM)

2. **How long does it usually take you to fall asleep each night?**
   Number of minutes: _______________ (e.g., 10 minutes)

3. **When did you usually wake up in the morning?**
   Usual wake-up time: ________________ (e.g., 7:30 AM)

4. **How many hours of actual sleep did you get each night during the past month? (This may differ from the time spent in bed)**
   Number of hours of sleep: ____________ (e.g., 6.5 hours)

5. **How often did you have trouble sleeping because you...**

S. No	Statements	Not during the past month	Less than once per week	Once or twice in the week	Three times or more In the week
1.	Could not sleep within 30 minutes				
2.	Woke up in the middle of the night or early morning				
3.	Had to get up to use the bathroom				
4.	Could not breathe comfortably				
5.	Coughed or snored loudly				
6.	Felt too cold				
7.	Felt too hot				
8.	Had bad dreams				
9.	Feeling pain				
10.	Other causes Please explain.....during the past month how many times have you had problems sleeping because of this?				

6. **How would you rate your overall sleep quality during the past month?**
- Very good
- Good
- Fairly bad
- Very bad

S. No	Statements	Not during the past month	Less than once per week	Once or twice in the week	Three times or more In the week
7.	**How often did you take medication, prescribed or over-the-counter, to help you sleep during the past month?**				
8.	**How often did you have trouble staying awake while driving, eating meals, or engaging in social activities during the past month?**				

9. **How much of a problem did you have with keeping up enough enthusiasm to get things done during the past month?**
   - No problem at all
   - Only a very slight problem
   - Somewhat of a problem
   - A very big problem

10. **Do you have a bed partner or share the room?**
   - No bed partner or room sharing
   - A partner in another room
   - Bed partner in the same room but not the same bed
   - Bed partner in the same bed

11. If you have a bed partner or share a room, ask him/her during the past month, how many times have you had:

S. No	Statements	Not during the past month	Less than once per week	Once or twice in the week	Three times or more In the week
1.	Snore loudly				
2.	A long pause in breathing during sleep				
3.	Restless legs while asleep (unstable)				
4.	Episodes of confusion during sleep				
5.	Any discomfort during sleep: Explain please				
